# Associations of cardiometabolic outcomes with indices of obesity in children aged 5 years and younger

**DOI:** 10.1371/journal.pone.0218816

**Published:** 2019-07-05

**Authors:** Koon K. Teo, Talha Rafiq, Sonia S. Anand, Karleen M. Schulze, Salim Yusuf, Sarah D. McDonald, Gita Wahi, Nora Abdalla, Dipika Desai, Stephanie A. Atkinson, Katherine M. Morrison

**Affiliations:** 1 Population Health Research Institute, Hamilton Health Sciences, McMaster University, Hamilton, Ontario, Canada; 2 Department of Medicine, McMaster University, Hamilton, Ontario, Canada; 3 Chanchlani Research Centre, McMaster University, Hamilton, Ontario, Canada; 4 Department of Health Research Methods, Evidence & Impact, McMaster University, Hamilton, Ontario, Canada; 5 Department of Obstetrics & Gynecology, McMaster University, Hamilton, Ontario, Canada; 6 Department of Radiology, McMaster University, Hamilton, Ontario, Canada; 7 Department of Pediatrics, McMaster University, Hamilton, Ontario, Canada; Cincinnati Children's, UNITED STATES

## Abstract

**Background:**

Childhood obesity is a world-wide concern due to its growing prevalence and association with cardiometabolic risk factors in childhood and subsequent adult cardiovascular disease. In young pre-school children, there is uncertainty regarding which of the commonly used anthropometric measures of childhood obesity is best associated with cardiometabolic risk factors. This study compared the utility of common measures used in identifying obesity in these young children.

**Methods:**

The four commonly used metrics for identifying obesity in children: body fat percentage ≥ 90^th^ percentile, waist circumference ≥ 90^th^ percentile, BMI z score > 2 SD and waist-to-height ratio (WHtR) ≥ 0.5, were measured in a cohort of children born singleton, at full term and followed from birth (n = 761) to 5 years of age (n = 513). The utility of each in identifying cardiometabolic risk factors (fasting lipid profile, fasting blood glucose and blood pressure) was examined.

**Results:**

At age 5 years, children with percent body fat ≥ 90^th^ percentile or waist circumference ≥ 90^th^ percentile, were associated with higher levels of triglycerides, glucose, and systolic and diastolic blood pressures than those < 90^th^ percentile, respectively. Such differences were not obvious at age 3 years or at birth. A BMI z-score > 2 SD was associated with higher levels of triglycerides and systolic and diastolic blood pressure but not glucose at age 5 years. Differences in HDL cholesterol, fasting glucose and systolic blood pressure were observed in children with BMI z score > 2 SD at age 3 years but not with the other indices of obesity. As almost all children had WHtR ≥ 0.5 at birth, ages 1 and 3 years, this measure could not differentiate increased cardiometabolic risk. At age 5 years, the differences were much more obvious, with significant differences in triglycerides and systolic and diastolic blood pressures between those with WHtR ≥ 0.5 and those with < 0.5.

**Conclusion:**

Each of the four commonly used measures of childhood obesity shows moderate associations with cardiometabolic risk factors at 5 years, with no advantage of one measure over the other. These associations were less consistent at 3 years of age or younger. These observations have not been reported previously.

## Introduction

Childhood obesity has risen dramatically and has become an increasing public health concern over the past few decades in both high and lower income countries [1]. This contributes to the epidemic of cardiovascular disease (CVD) in adult life [2]. Obesity is characterized by excess body adiposity, and existing studies show that childhood obesity is often associated with clustering of cardiometabolic risk factors in childhood and greater risk of subsequent CVD in adulthood [3]. Some studies have also shown that obesity in childhood tracks into adulthood suggesting an early onset and greater likelihood of CVD [4]. Despite this high prevalence of obesity, evaluation of adiposity to detect obesity is not done consistently in clinical practice [5].

A variety of methods are available for assessing obesity by researchers and clinicians. Rather than a direct estimate of the adiposity, it is usually derived from anthropometric measurements including weight, waist circumference (WC) and height [6]. Indices of obesity such as body mass index (BMI), or waist to height ratio (WHtR) can be derived from these measurements. The challenge is to decide which of these indices of obesity is appropriate to use in predicting the presence of cardiometabolic risk factors among very young individuals. In adults, with their grown height remaining constant, changes in weight and WC would reasonably reflect changes in soft adipose or non-adipose tissues. However, in the newborn and young child, rapid growth results in accompanying but non-uniform changes in weight, height and WC from which the indices of obesity are derived, and since these growths occur at different rates in the young children, the assumptions that were made in deriving the indexes of obesity in adults such as the BMI or WHtR may no longer hold. As a result, there is less certainty about which of the commonly used indices would be suitable and more appropriate for assessment of health consequences associated with obesity in children 5 years and younger. Simultaneously, children may also experience changes in cardiometabolic risk factors with growth, potentially shifting the strength of association between each index of obesity and risk factors. Therefore, it is uncertain whether a young child identified as being overweight or obese by any one of the indices of obesity has a greater likelihood of association with cardiometabolic risk factors. Comparison of the utility of these measures of obesity and their association with cardiometabolic risk factors have not been done previously.

To address this uncertainty, in this study we determined the prevalence of overweight and obesity in a cohort of children from birth to 5 years of age, using three common and easily obtained indices of obesity: BMI, WC and WHtR derived from anthropometric measures. In addition, the triceps and scapular skin fold thickness was measured and used to estimate body adiposity [7]. In order to determine the age at which the association between each of the indices of obesity and cardiometabolic risk factors become obvious and persistent, we explored the associations between each of these indices of obesity and cardiometabolic risk factors in a cohort of children between birth and 5 years of age.

## Methods

The data for this analysis were drawn from a longitudinal birth cohort study—the Family Atherosclerosis Monitoring in early Life (FAMILY) study, for which the protocol and methodology have been published previously [8,9]. The study was approved by the Research Ethics Boards at the participating hospitals (Hamilton Health Sciences, St. Joseph’s Hospital in Hamilton, Ontario, and Joseph Brant Memorial Hospital, Burlington, Ontario). Briefly, all consenting expectant mothers who planned to have their babies delivered at one of the three recruiting hospitals in the Hamilton area in Southern Ontario were approached for the study. Mothers were enrolled when they were between 24 and 36 weeks of gestation. At the enrollment visit, the mother’s demographic information, family socioeconomic status (education and household income), health and nutrition, medical and pregnancy history and CVD risk factors were assessed by a series of questionnaires. Physical examination and blood tests for fasting lipids and glucose levels were also conducted. A 75 g oral glucose tolerance test was done (for those not known to have diabetes) to evaluate glycemic status. Following the birth-visit, the families were contacted by telephone at 6 months, and then followed up with clinic visits at 1, 2, 3, and 5-years.

### Measurements

Birth data including information on maternal labour, delivery, and pregnancy history were abstracted from medical charts. Birth visits for the newborn were conducted within 3 days of birth, and a home visit was made within 1 week of birth among the few individuals who were discharged prior to the birth visit. Subsequent visits were scheduled at or near the child’s 1st, 2nd, 3rd and 5th year birthdays.

Body weight was measured using a standard hospital electronic pediatric scale to the nearest 10 grams, and while length of children below 2 years was measured using a length board to the nearest 0.1 cm (O’Leary Length Board, Ellard Instrumentation Ltd), height was measured to the nearest 0.5 cm using a stadiometer at the 3- and 5-year follow-up visits (Ellard Stadiometer, Ellard Instrumentation Ltd). All measurement procedures followed standardized protocols. Throughout this manuscript we will use the term height for both the length and height. WC was measured to the nearest 0.1 cm at a point half way between the lower border of the rib cage and superior border of the iliac crest, using the OHAUS (Florham Park, NJ) non-stretchable tape, with attached pressure gauge to ensure a standardized tension. Blood pressure was measured using a calibrated automatic measuring device (Dinamap Pro100 V2, GE Medical Systems, Tampa, Florida) using an appropriate cuff size based on the arm circumference. Two independent sequential measures were taken at 5-minute intervals and were averaged. Sex and height adjusted z-scores were computed for blood pressure at each age group and the adjusted z-scores were used in the analysis. Blood samples for glucose and lipid levels were obtained from children at three different time periods: from cord blood at birth and fasting samples at the 3rd- and 5th year follow-up visits, and were analyzed at the McMaster Children’s Hospital and Hamilton Health Sciences Central Research Laboratories following standardized analysis protocols described previously [8,9].

### Indices of obesity

Body fat percentage from skin fold thickness: The sum of triceps and subscapular skinfold thickness from each child were recorded at birth and during each follow up visits. Percent body fat for each child was calculated by using the formula by Slaughter et al [7]. Obesity was defined as sex-specific percent body fat ≥ 90 percentile [10].

Body Mass Index (BMI): This index was calculated by dividing weight in kilograms over height in meter squared (kg/m^2^) and each BMI value was transformed into percentiles for age and sex. Obesity was defined using the age- and sex-specific classification established by the Center for Disease Control and Prevention (CDC) and the American Heart Association, where overweight was defined as BMI≥85^th^ and less than 95^th^ percentile, class I obesity was defined as BMI≥95^th^ percentile and less than 120% of the 95^th^ percentile, class II obesity was defined as BMI≥120% of the 95^th^ percentile and less than 140% of the 95^th^ percentile, and class III obesity was defined as BMI≥140% of the 95^th^ percentile [11–13]. In our sample, no participants aged 3 years were classified as class II or class III obese, and no participants aged 5 years were classified as class III obese. BMI measures were not analyzed for children at birth as the use of BMI-for-age growth chart is not recommended for children younger than 2 years [14].

Waist Circumference (WC): This index was presented in percentiles and was derived from the criteria established by Ahren et al [15], where obesity was classified as sex-specific WC ≥ 90 percentile, for children 3 years and older.

Waist circumference to height ratio (WHtR): This was the ratio of WC (cm) to height (cm). The criteria for obesity for adolescents and adults was WHtR ≥ 0.5 and this cut-off was used as an indicator of obesity in this study since no criteria have been established for pre-school children [16].

### Statistical analysis

Descriptive statistics for anthropometrics, blood pressure, and laboratory measurement are presented as mean and standard deviation for all children from birth to 5 years of age. We conducted univariate analysis to test the association between cardiometabolic risk factors and covariates including ethnicity, minutes spent in performing physical activity, annual household income, gestational age, and maternal smoking during pregnancy at the different ages and did not find them to be consistently and significantly associated with the outcomes. Next, we examined the distribution of obesity using the four indices across the five follow-up visits. A series of analyses of covariance (ANCOVA) were performed to examine the relationship between obesity and each measure of cardiometabolic risk (total cholesterol, triglycerides, HDL and LDL cholesterol, fasting plasma glucose, systolic and diastolic blood pressure, and triglyceride levels), after adjusting for age, sex, ethnicity, annual household income, gestational age, maternal smoking during pregnancy, birth weight, and physical activity levels (at 3- and 5-years).

Next, we tested these models using mixed-effects modelling to longitudinally examine the relationship between each index of obesity and the cardiometabolic risk factors over time after adjusting for all previously mentioned covariates (S1 Table). Interaction between time and obesity index was tested to examine if cardiometabolic risk factors changed over the follow-up time period among children who were identified be obese and those who were not obese. Statistical significance for a two-tailed test was set at p≤0.05 and all analysis were performed using SAS 9.4 (SAS Institute, Cary, NC).

## Results

Recruitment of family members took place between April 2004 and June 2009. At baseline, a total of 857 families, with 901 babies, had been enrolled. Over 85% of these families were of European ethnicity. For the current analysis, we included singleton children born at full term with follow-up data for visits which occurred no more than 10 months past their birthday for the year of visit. This study included 761 children at baseline (birth), 685 at year 1, 652 at year 2, 604 at year 3, and 513 singleton children at year 5 who had complete data. Descriptive characteristics of the participants at each assessment are reported in Table 1.

**Table 1:** Descriptive characteristics of mothers and children over the study period.

Characteristics	N	Birth	3-year	5-year
Age (years), mean (SD)		–	3.1 (0.2)	5.1 (0.2)
Sex, n (%)	761			
Male		389 (51.1)	315 (50.6)	261 (50.3)
Female		372 (48.9)	307 (49.4)	258 (49.7)
Gestational age (weeks), mean (SD)	761	39.5 (1.2)	–	–
Breastfeeding of ≤6 months, n (%)	724	196 (27.1)	–	–
Smoked during pregnancy, n (%)	737	103 (14.0)	–	–
Annual household income (<$50,000), n (%)	737	168 (22.8)	–	–
Birth weight (kg), mean (SD)	761	3.53 (0.48)	–	–
Family history of CVD, n (%)	757	147 (19.42)	–	–
Birth length (cm), mean (SD)	736	50.2 (2.1)	–	–
Level of activity of child (week), n (%)				
Inactive/very inactive			3 (0.6)	5 (0.8)
Somewhat inactive			38 (7.4)	15 (2.5)
Somewhat active			233 (45.5)	181 (30.0)
Active/very active			238 (46.5)	403 (66.7)

### Anthropometrics, laboratory measures and blood pressure

Table 2 summarizes the laboratory, anthropometric, and blood pressure measurements of children from birth to 5 years of age. As can be derived from data in Table 2, there are differences in rates of growth depending on the age and anthropometrics. On average, height and WC increased rapidly from birth to 1 year of age, then more gradually until 5 years of age. This difference was further accentuated from 1 to 5 years of age, with height increasing more rapidly than WC such that by 5 years of age, height had increased three times more than WC (Table 2).

**Table 2:** Anthropometrics, blood pressure, and laboratory measurements on the full term, singleton children from birth to age 5 visit.

	Birth		Year 1		Year 2		Year 3		Year 5	
	N	Mean (SD)	N	Mean (SD)	N	Mean (SD)	N	Mean (SD)	N	Mean (SD)
**Anthropometrics**										
Weight (kg)	761	3.5 (0.5)	685	10.1 (1.3)	652	12.8 (1.5)	604	15.0 (1.8)	513	19.5 (2.8)
Weight for age (WHO Z)	761	0.45 (0.97)	685	0.49 (1.01)	652	0.51 (0.92)	604	0.32 (0.90)	513	0.30 (0.93)
Length/Height (cm)	736	50.2 (2.1)	679	75.9 (3.3)	645	88.2 (3.5)	598	95.9 (3.9)	513	110.7 (4.7)
Height for age (WHO Z)	736	0.37 (1.13)	679	0.02 (1.09)	645	0.29 (1.03)	598	-0.06 (0.96)	513	0.08 (0.96)
Waist circumference (cm)	709	29.9 (2.4)	680	44.5 (3.1)	646	47.4 (3.1)	593	48.3 (3.1)	512	51.7 (4.1)
Hip circumference (cm)	734	28.2 (2.4)	684	45.0 (3.3)	640	48.7 (3.2)	596	51.3 (3.2)	512	57.7 (4.1)
**Blood pressure**										
Systolic (mm Hg)	632	69.6 (9.6)	466	96.0 (11.4)	536	96.9 (10.7)	568	95.6 (8.9)	508	99.4 (8.4)
Diastolic (mm Hg)	632	39.1 (8.1)	464	59.6 (7.6)	536	60.7 (7.9)	568	60.2 (5.9)	508	60.2 (5.5)
**Laboratory Measures**										
Total cholesterol (mmol/L)	588	1.64 (0.42)	-	-	-	-	552	4.03 (0.68)	459	4.09 (0.70)
HDL cholesterol (mmol/L)	587	0.78 (0.27)	-	-	-	-	552	1.32 (0.29)	459	1.42 (0.32)
LDL cholesterol (mmol/L)	588	0.68 (0.26)	-	-	-	-	552	2.39 (0.61)	459	2.36 (0.65)
Triglycerides (mmol/L)	587	0.38 (0.22)	-	-	-	-	552	0.68 (0.28)	459	0.70 (0.31)
Fasting Glucose (mmol/L)	589	4.35 (0.94)	-	-	-	-	551	4.49 (0.42)	459	4.63 (0.36)

Birth blood samples from cord blood. No blood samples drawn at year 1 and year 2. HDL—High-density lipoproteins, LDL—Low-density lipoprotein, SBP—Systolic blood pressure, DBP—Diastolic blood pressure, SD—Standard deviation.

Both systolic and diastolic blood pressures increased rapidly by 1.4 to 1.5-fold over the first year after birth, and then in smaller increments for each of the next 4 years. These changes are typical for most children during the first 5 years of life. At birth, mean plasma LDL cholesterol and HDL cholesterol concentrations were similar and were low by adult standards (Table 2). Over the 5 years, plasma LDL cholesterol had increased 3.5-fold and HDL cholesterol by 1.8-fold. Triglyceride levels increased by 1.8-fold over the 5 years. Fasting glucose remained relatively stable over the 5 years.

### Indices of obesity

Table 3 shows the average score for each index of obesity by age, from birth to age 5 years. *Skin fold thickness* increased from 10.4 mm at birth to 19.0 mm at year 1, after which it remained stable around 18.5 mm up until year 5. Percent body fat, derived from the sum of triceps and subscapular skin fold thickness [7] also followed a similar pattern where it increased from 9.9% at birth to 18.1% at 1 year, and then remained constant around 17.6% in subsequent years. The proportions of individuals with percent body fat ≥ 90^th^ percentile was 11.2% at birth and then varied between 10.2% and 10.5% in later years. The actual percent body fat in this obese subgroup increased from 14.5% at birth to 24.8% at 1 year and remained more or less constant after that. Approximately, 7.2% and 11.5% of children had WC ≥ 90^th^ percentile at 3 and 5 years of age, respectively; mean WC in these subgroups was 54.9 (SD 1.8) cm and 59.5 (SD 4.4) cm, respectively. Around 80% of children at 3 and 5 years were classified as having a healthy BMI status, approximately 13% of children were identified as being overweight, and 6% as being class I obese. No participants were identified as class II obese at 3 years, but 1.2% were identified as class II obese at 5 years. The cut-off for *WHtR* of greater than or equal to 0.5 for identifying obesity in older children and adults, was present in nearly all children from birth to 2 year of age. With growth, there is proportionally greater increase in height than waist, and as a result, the proportion of individuals with WHtR ≥ 0.5 had declined to 14.4% by year 5.

**Table 3:** Markers and prevalence of obesity by age.

	Birth	Year 1	Year 2	Year 3	Year 5
**Skinfold thickness** (mm)	10.4 (2.5)	19.0 (4.4)	18.5 (4.4)	18.7 (4.5)	18.7 (5.9)
Percent body fat (PBF)*	9.9 (2.6)	18.1 (3.7)	17.6 (3.7)	17.7 (3.8)	17.6 (4.6)
No. (%) with PBF ≥ 90^th^ percentile	75 (11.2)	71 (10.5)	65 (10.2)	61 (10.4)	52 (10.3)
Mean (SD) BF in obese subgroup	14.5 (1.9)	24.8 (1.5)	24.5 (1.9)	24.6 (2.0)	26.5 (3.4)
**Mean WC** (SD) cm	29.9 (2.4)	44.5 (3.1)	47.4 (3.1)	48.3 (3.1)	51.7 (4.1)
No. (%) ≥ 90 percentile**	NA	NA	NA	43 (7.2)	59 (11.5)
Mean (SD) WC in obese subgroup				54.9 (1.8)	59.5 (4.4)
**Mean BMI**	NA^†^	NA^†^	NA^†^	16.2 (1.2)	15.9 (1.6)
Mean percentile for BMI for age	NA^†^	NA^†^	NA^†^	56.4 (27.6)	57.8 (27.7)
No. (%) for obesity status	NA^†^	NA^†^	NA^†^		
Healthy				485 (81.1)	407 (79.3)
Overweight				77 (12.9)	69 (13.5)
Class I obesity				36 (6.0)	31 (6.0)
Class II obesity					6 (1.2)
**Mean WHtR (SD)**	0.595 (0.042)	0.586 (0.038)	0.538 (0.033)	0.503 (0.030)	0.468 (0.034)
Proportion WHtR ≥ 0.50	99.4% (690/694)	99.0% (668/675)	90.0% (576/640)	51.5% (304/590)	14.4% (74/512)
Mean (SD) WHtR in subgroup	0.596 (0.041)	0.587 (0.037)	0.544 (0.028)	0.526 (0.022)	0.528 (0.032)

WC—Waist circumference, BMI—Body Mass Index, WHtR—Waist circumference-to-height ratio, SD—Standard deviation.

*Based on Slaughter et al [7];

**Ahren’s et al [15] criteria (available age 3+);

^†^use of BMI-for-age growth chart is not recommended for children younger than 2 years [14].

### Association with cardiometabolic risk factors

The results examining the association of percent body fat, waist circumference, BMI z-scores, and WHtR with cardiometabolic risk factors are presented in Tables 4–7, respectively.

**Table 4:** Association of percent body fat (from skin fold thickness) with cardiometabolic risk factors, age & sex adjusted, at birth, and ages 3 and 5 years.

	Birth Measures			Year 3 Measures			Year 5 Measures		
	BF ≥90%	BF <90%	P-value	BF ≥90%	BF <90%	P-value	BF ≥90%	BF <90%	P-value
N	75	597	.	61	527	.	52	455	.
Total Cholesterol mmol/L, mean (SE)	1.67 (0.06)	1.63 (0.02)	0.51	3.97 (0.10)	4.03 (0.03)	0.55	4.19 (0.10)	4.07 (0.04)	0.27
HDL Cholesterol mmol/L, mean (SE)	0.75 (0.04)	0.79 (0.01)	0.29	1.27 (0.04)	1.32 (0.01)	0.23	1.43 (0.05)	1.41 (0.02)	0.68
LDL Cholesterol mmol/L, mean (SE)	0.73 (0.04)	0.67 (0.01)	0.18	2.39 (0.09)	2.39 (0.03)	0.99	2.40 (0.10)	2.34 (0.03)	0.56
Triglycerides mmol/L, mean (SE)	0.40 (0.03)	0.38 (0.01)	0.54	0.70 (0.04)	0.68 (0.01)	0.73	0.80 (0.05)	0.69 (0.02)	**0.03**
Fasting Glucose mmol/L, mean (SE)	4.05 (0.13)	4.37 (0.04)	**0.02**	4.60 (0.06)	4.47 (0.02)	**0.04**	4.75 (0.05)	4.61 (0.02)	**0.02**
SBP-z, mean (SE)	-0.02 (0.13)	0.01 (0.04)	0.80	0.05 (0.14)	-0.04 (0.05)	0.51	0.55 (0.14)	-0.08 (0.05)	**< .0001**
DBP-z, mean (SE)	-0.11 (0.13)	0.01 (0.04)	0.40	0.05 (0.14)	-0.02 (0.05)	0.62	0.39 (0.14)	-0.07 (0.05)	**0.003**

HDL—High-density lipoproteins, LDL—Low-density lipoprotein, SBP-z—Sex and height adjusted z-scores for systolic blood pressure, DBP-z—Sex and height adjusted z-scores for diastolic blood pressure. Significant differences are indicated in bold.

All analysis were adjusted for age, sex, ethnicity, annual household income, gestational age, maternal smoking during pregnancy, birth weight, and physical activity levels (at 3- and 5-years).

**Table 5:** Association of waist circumference (WC) with cardiometabolic risk factors, age & sex adjusted, at ages 3 and 5 years.

	Year 3 Measures			Year 5 Measures		
	WC ≥90%	WC <90%	P-value	WC ≥90%	WC <90%	P-value
N	43	550	.	56	447	.
Total Cholesterol mmol/L, mean (SE)	4.13 (0.12)	4.02 (0.03)	0.22	4.14 (0.10)	4.07 (0.04)	0.52
HDL Cholesterol mmol/L, mean (SE)	1.31 (0.05)	1.32 (0.01)	0.89	1.37 (0.05)	1.42 (0.02)	0.32
LDL Cholesterol mmol/L, mean (SE)	2.49 (0.11)	2.39 (0.03)	0.39	2.39 (0.09)	2.34 (0.03)	0.59
Triglycerides mmol/L, mean (SE)	0.74 (0.05)	0.68 (0.01)	0.29	0.89 (0.04)	0.69 (0.02)	**0.002**
Fasting Glucose mmol/L, mean (SE)	4.69 (0.07)	4.47 (0.02)	**0.005**	4.78 (0.05)	4.61 (0.02)	**0.002**
SBP-z, mean (SE)	0.08 (0.16)	-0.03 (0.04)	0.53	0.35 (0.13)	-0.06 (0.05)	**0.005**
DBP-z, mean (SE)	0.28 (0.17)	-0.03 (0.05)	0.07	0.19 (0.14)	-0.04 (0.05)	0.10

HDL—High-density lipoproteins, LDL—Low-density lipoprotein, SBP-z—Sex and height adjusted z-scores for systolic blood pressure, DBP-z—Sex and height adjusted z-scores for diastolic blood pressure. Significant differences are indicated in bold.

All analysis were adjusted for age, sex, ethnicity, annual household income, gestational age, maternal smoking during pregnancy, birth weight, and physical activity levels (at 3- and 5-years).

**Table 6:** Association of body mass index (BMI) percentiles for age classification with cardiometabolic risk factors, age & sex adjusted, at ages 3 and 5 years.

	Year 3 Measures				Year 5 Measures				
	Normal	Overweight	Class I Obese	p-value	Normal	Overweight	Class I Obese	Class II Obese	p-value
N	485	77	36		407	69	31	6	
Total Cholesterol mmol/L, mean (SE)	4.03 (0.03)	3.96 (0.09)	4.06 (0.13)	0.71	4.08 (0.04)	4.07 (0.10)	4.14 (0.13)	3.76 (0.32)	0.75
HDL mmol/L, mean (SE)	1.32 (0.01)	1.27 (0.04)	1.38 (0.05)	0.25	1.41 (0.02)	1.44 (0.04)	1.45 (0.06)	1.03 (0.14)	**0.04**
LDL mmol/L, mean (SE)	2.40 (0.03)	2.38 (0.08)	2.39 (0.11)	0.98	2.35 (0.03)	2.34 (0.09)	2.32 (0.12)	2.18 (0.29)	0.95
Triglycerides mmol/L, mean (SE)	0.69 (0.01)	0.67 (0.04)	0.70 (0.05)	0.85	0.70 (0.02)	0.63 (0.04)	0.80 (0.06)	1.19 (0.14)	**0.001**
Fasting Glucose mmol/L, mean (SE)	4.46 (0.02)	4.62 (0.05)	4.62 (0.08)	**0.005**	4.60 (0.02)	4.74 (0.05)	4.73 (0.07)	4.91 (0.16)	**0.006**
SBP-z, mean (SE)	-0.06 (0.12)	-0.003 (0.12)	0.40 (0.18)	**0.045**	-0.11 (0.05)	0.27 (0.13)	0.60 (0.18)	1.22 (0.41)	**< .0001**
DBP-z, mean (SE)	-0.06 (0.05)	0.23 (0.12)	0.20 (0.18)	**0.047**	-0.07 (0.05)	0.07 (0.13)	0.58 (0.19)	0.05 (0.42)	**0.008**

HDL—High-density lipoproteins, LDL—Low-density lipoprotein, SBP-z—Sex and height adjusted z-scores for systolic blood pressure, DBP-z—Sex and height adjusted z-scores for diastolic blood pressure. Significant differences are indicated in bold.

All analysis were adjusted for age, sex, ethnicity, annual household income, gestational age, maternal smoking during pregnancy, birth weight, and physical activity levels (at 3- and 5-years).

**Table 7:** Association of waist-to-height ratio (WHtR) with cardiometabolic risk factors, age & sex adjusted, at birth, and ages 3 and 5 years.

	Birth Measures			Year 3 Measures			Year 5 Measures		
	WHtR ≥.5	WHtR < .5	P-value	WHtR ≥.5	WHtR < .5	P-value	WHtR ≥.5	WHtR < .5	P-value
N	694	5	.	304	286	.	74	438	.
Total Cholesterol mmol/L, mean (SE)	1.64 (0.02)	1.85 (0.19)	0.27	4.01 (0.04)	4.04 (0.04)	0.67	4.11 (0.09)	4.08 (0.04)	0.72
HDL mmol/L, mean (SE)	0.79 (0.01)	0.83 (0.12)	0.76	1.28 (0.02)	1.35 (0.02)	**0.01**	1.38 (0.04)	1.42 (0.02)	0.35
LDL mmol/L, mean (SE)	0.68 (0.01)	0.79 (0.12)	0.09	2.41 (0.04)	2.38 (0.04)	0.63	2.38 (0.08)	2.34 (0.03)	0.68
Triglycerides mmol/L, mean (SE)	0.38 (0.01)	0.49 (0.09)	0.23	0.71 (0.02)	0.66 (0.02)	0.08	0.80 (0.04)	0.69 (0.02)	**0.009**
Fasting Glucose mmol/L, mean (SE)	4.36 (0.04)	4.27 (0.48)	0.86	4.50 (0.03)	4.47 (0.03)	0.32	4.67 (0.05)	4.62 (0.02)	0.39
SBP-z, mean (SE)	0.01 (0.04)	-0.07 (0.45)	0.87	0.07 (0.06)	-0.11 (0.06)	**0.04**	0.30 (0.12)	-0.06 (0.05)	**0.001**
DBP-z, mean (SE)	-0.01 (0.04)	0.05 (0.45)	0.90	0.08 (0.06)	-0.10 (0.06)	**0.049**	0.37 (0.12)	-0.08 (0.05)	**0.001**

HDL—High-density lipoproteins, LDL—Low-density lipoprotein, SBP-z—Sex and height adjusted z-scores for systolic blood pressure, DBP-z—Sex and height adjusted z-scores for diastolic blood pressure. Significant differences are indicated in bold.

All analysis were adjusted for age, sex, ethnicity, annual household income, gestational age, maternal smoking during pregnancy, birth weight, and physical activity levels (at 3- and 5-years).

#### Percent body fat

At birth, 3 years, and 5 years of age, children with body fat percentage ≥ 90^th^ percentile had higher fasting glucose than those with body fat < 90^th^ percentile. In addition to persistently higher levels of fasting glucose, children with body fat percentage ≥ 90^th^ percentile also had higher levels of triglycerides and systolic and diastolic blood pressures by the age of 5 years.

#### Waist circumference

At age 3 years, children with WC ≥ 90^th^ percentile had higher fasting glucose than those with WC < 90^th^ percentile. Meanwhile at 5 years, children with WC ≥ 90^th^ percentile had higher levels of triglycerides, fasting glucose, and systolic blood pressure compared to those with WC < 90^th^ percentile.

#### BMI z-scores

At 3 years of age, children who were identified as overweight or class I obese had a slightly higher fasting glucose and diastolic blood pressure than children who had a normal body weight status. In this age group, systolic blood pressure was significantly elevated among those with class I obesity compared to the normal weight and overweight groups. At 5 years of age, glucose levels were higher in the overweight and class I obesity groups, and even more elevated in the class II obesity group, suggesting a positive association between glucose levels and the severity of obesity. A positive association between severity of obesity and systolic blood pressure was also found. In children with normal weight status, systolic blood pressure was lower than the gender and height adjusted average (z-score = -0.12), and then increased with severity of obesity (overweight: z-score = 0.32, class I obesity: z-score = 0.64; class II obesity: z-score = 1.29). Higher diastolic blood pressure was also evident among children classified into the overweight, class I, and class II obesity groups compared to the normal weight group. Additionally, children in class II obesity group had decreased HDL cholesterol and elevated triglyceride levels.

#### WHtR

In this study, nearly all newborns had WHtR ≥ 0.5 up to 2 years of age, therefore no difference in cardiometabolic factors could be observed. At 3 years, there were a modest increase in systolic and diastolic blood pressure and decrease in HDL cholesterol. However, at 5 years of age, the differences were much more obvious, especially for systolic and diastolic blood pressures. Children with WHtR ≥ 0.5 had higher triglycerides, and systolic and diastolic blood pressures than those with WHtR < 0.5.

Mixed-effects models were tested to examine the relationship between cardiometabolic risk factors and obesity indices over time, after adjusting for covariates. The results are presented in S1 Table. The results show that systolic blood pressure increases much more rapidly over time from 3 to 5 years among children who were in the top 10 percentile of body fat compared to children with body fat less than the 90^th^ percentile. Additionally, children who were identified to be obese using the WHtR experienced an increase in total cholesterol levels over a period of 3 to 5 years when compared to children who were non-obese. With the exception of these findings, overall, results from mixed-effects models did not indicate any significant change in cardiometabolic risk factors over the study period in obese children compared to non-obese children.

## Discussion

Increasing epidemiological evidence suggests that obesity in childhood is associated with various cardiometabolic risk factors including elevated blood pressure, dyslipidemia, insulin resistance, dysglycemia and type-2 diabetes [17–20], which in turn increases the risk of developing CVD in adulthood. Our study identified that childhood obesity could be associated with cardiometabolic risk factors at the young age of 5 years. In this cohort of children, all four measures of obesity that we employed were associated with higher cardiometabolic risk, including triglycerides, and systolic and diastolic blood pressure compared to those who were not obese. Moreover, children with increased body fat percentage (≥ 90^th^ percentile) estimated from sum of skin thickness, elevated WC (≥ 90^th^), and class II obesity as classified by the BMI exhibited higher fasting glucose levels at the age of 5 years. The observed association between obesity and cardiometabolic risk factors, irrespective of how obesity was measured, are consistent with previous reports on older children [21–25]. The observations in this study were made exclusively in a cohort of 5 year old children, although some differences in fasting glucose levels and systolic blood pressure were also evident at of 3 years. There was no significant change in cardiometabolic risk factors over time between obese and non-obese groups (with the exception of an interaction between time and percentage body fat on systolic blood pressure, and between time and WHtR on total cholesterol levels).

Owing to its simplicity and routine measurements of weight and height, BMI is widely considered to be a common and useful index of obesity in children and adolescents, and is typically based on age- and sex-dependent cut-offs for standardization [12,13]. In this study, we classified BMI into several categories of obesity status. Our findings of the positive association between severity of obesity and fasting glucose, systolic blood pressures, and triglyceride levels are consistent with the results reported by another study conducted among children and young adults 3–19 years of age [26]. Although the difference in risk factors between obese and non-obese groups may not be clinically significant at 5 years of age, the findings are still clinically important.

Studies have shown childhood obesity to be a risk factor for obesity later in life [27,28]. There is also evidence suggesting that the atherosclerotic process may begin in childhood [29], and without prevention and intervention measures, the presence of elevated cardiometabolic risk factors can speed up the atherosclerotic process and increase the risk of CVD. In addition, obesity during childhood is also associated with increased risk of premature mortality and mortality in adulthood [30,31].

While the observed cardiometabolic profile is largely consistent across the different measures of obesity, there are a number of important nuances in assessment of obesity in these children. WC is a widely known measure for assessing abdominal (central) obesity and is associated with poor cardiometabolic health in children 5 years of age and older [32]. Most studies in children using WC have based their assessment on the criteria by the International Diabetes Federation (IDF), where a ≥ 90^th^ percentile cut-off is considered obese [33]. WC is dependent on age and gender cut-offs [16], and though it is also dependent on height, it does not take height into consideration. As a result, central obesity may possibly be overestimated in very tall, and underestimated in very short individuals.

Another index that has recently gained attention as a measure of central adiposity in children population is WHtR. A commonly accepted cut-off of ≥ 0.5 is used to identify obesity in children and adults [16]. Recent studies have suggested that WHtR may be equivalent or a slightly better predictor of cardiometabolic health than BMI in children and adolescents [21,34]. However, the use of WHtR is not recommended in very young children [35] due to the fact that WHtR cut-off of 0.5 tends to overestimate obesity in this sub-group. This is evident from our own results that over ~90% of children at the age of 2 years and ~50% at the age of 3 years had WHtR ≥ 0.5. However, at 5 years of age, the proportion of children identified as obese using WHtR (14.4%) is similar to those obtained using other measures, and is consistent with a previous study [36].

The measure of percent body fat using skin-fold thickness is a well-established and valid index of obesity in the children population [37]. Skinfolds are the double layer of skin and subcutaneous fat that is measured with standardized calipers at selected sites, most commonly, triceps, subscapular, iliac, and bicep [38]. Most studies in children estimate percent body fat using skinfold thickness using the Slaughter equation [7]. To its advantage, this index is non-invasive and specific to subcutaneous fat, however, some experts in the field do not recommend the routine clinical use of this index [38]. The concern is that reliability and reproducibility of skinfold measurement is much lower than for height and weight [39], even among well-trained operators, and most clinicians and community-setting personnel do not have adequate experience to reliably measure skin-fold thickness [37]. Finally, while optimal reference percentiles are available for children and adults [7,40,41], no such percentile cut-offs have been defined in preschool children. In this study we used the 90^th^ percentile cut off.

Taken overall, each measure of obesity has strengths and limitations, and the limitations associated with these indices of obesity may at least partly explain why the associations between cardiometabolic risk factors and obesity were not consistently observed at birth and 3 years of age. In these young children, along with the different rates of growth among individual children, there is less certainty about the definitions for obesity based on the diagnostic cut-offs for each of the measures of obesity. Moreover, the levels of the individual cardiometabolic risk factors vary with age and growth and what could be defined as adverse levels remains unclear [42] until about 5 years of age.

Previous studies have addressed similar questions but not exclusively in children aged 5 years or younger. Mokha et al found that among normal weight children from 4 to 18 years of age, those with central obesity had adverse levels of cardiometabolic risk factors, compared to those without central obesity (5.9% vs. 0.3%) [43]. Meanwhile, among overweight or obese children, those with central obesity exhibited much greater adverse cardiometabolic risk factors (21.3% vs. 0%) compared to those without central obesity. A recent systematic review concluded that abdominal obesity in children (WC and WHtR) is positively associated with cardiometabolic risk factors [44]. While this systematic review suggested WHtR to be equally effective as WC in predicting cardiometabolic health, the age of participants from included studies ranged from 6 to 18 years, older than our study participants. It is likely that WHtR cut-off of ≥ 0.5 is not applicable in very young children, but may be a more useful marker of cardiometabolic factors for children 5 years and older.

### Strengths and weaknesses

By systematically collecting detailed data on children’s characteristics from birth to age 5 years in a birth cohort, we were able to examine the association between cardiometabolic risk factors and general and central abdominal obesity in these pre-school children. By using the accepted definitions for 4 commonly used indices of obesity, we have moderate ability to identify young children at risk of developing higher levels of cardiometabolic risk factors than those without excess obesity. A limitation of this study is that while obesity at five years of age was associated with higher cardiometabolic profiles, the levels at which the risk factors are physiologically detrimental are yet to be defined. However, except for triglycerides, levels of cholesterol and its subfractions were not differentiated between children identified as obese as compared to those not obese. Another limitation is that, of the 761 singletons at birth, one third did not have adequate data to be included in the analyses at age 5 years. This attrition rate is comparable to most other birth cohort studies due to families moving and other social reasons. Having said this, in our study there was no significant difference in the mean scores for exposure and outcome variables between those participants who completed the study and those who did not. Finally, given the largely homogenous European background of the study population, the applicability of our findings to other ethnic groups is uncertain and should be evaluated in future studies.

## Conclusion

Each of the four commonly used measures of obesity in children: percent body fat, WHtR, WC and BMI show moderate associations with cardiometabolic risk factors at 5 years but not with consistency at 3 years of age or younger. Such simultaneous findings have not been reported previously in the same cohort of children. Continued follow-up of these children and relating the measures of obesity to long term outcomes will help in the understanding of development of cardiometabolic risk factors and CVD.

## Supporting information

S1 File Dataset for analysis. (XLSX)

S1 Table Mixed-effects models predicting cardiometabolic risk factors by indices of obesity from 3 to 5 years. (DOCX)
