# Supplementary material for: Associations of cardiometabolic outcomes with indices of obesity in children aged 5 years and younger
Source: PLoS One. 2019 Jul 5;14(7):e0218816. doi: 10.1371/journal.pone.0218816 (PMC6611590; doi:10.1371/journal.pone.0218816)
Supplement: S1 Table — (DOCX) [file pone.0218816.s002.docx]

**S1 Table: Mixed-effects models predicting cardiometabolic risk factors by indices of obesity from 3 to 5 years**

|  | **Total Cholesterol** | | **HDL** | | **LDL** | | **Fasting Glucose** | | **SBP** | | **DBP** | | **Triglycerides** | |
| --- | --- | --- | --- | --- | --- | --- | --- | --- | --- | --- | --- | --- | --- | --- |
|  | **E** | **p** | **E** | **p** | **E** | **p** | **E** | **p** | **E** | **p** | **E** | **p** | **E** | **p** |
| **WHtR** |  | |  | | | | | | | | | | | |
| Intercept | 3.6502 | 0.0002 | 1.9094 | <.0001 | 1.3083 | 0.1439 | 4.7214 | <.0001 | 1.6476 | 0.1758 | 2.3137 | 0.0587 | 1.0659 | 0.0048 |
| Time (5 y) | -0.00622 | 0.8632 | 0.08525 | <.0001 | -0.1052 | 0.0010 | 0.1650 | <.0001 | 0.03212 | 0.6540 | 0.01256 | 0.8637 | 0.02171 | 0.3190 |
| WHtR (≥.50) | -0.00688 | 0.8835 | -0.01958 | 0.3972 | -0.00013 | 0.9976 | 0.03545 | 0.3042 | 0.1541 | 0.0664 | 0.1715 | 0.0445 | 0.04070 | 0.1095 |
| WHtR*time | 0.1624 | **0.0339** | 0.02166 | 0.5772 | 0.1134 | 0.0910 | -0.01643 | 0.7903 | 0.1398 | 0.3274 | 0.2256 | 0.1214 | 0.05999 | 0.1812 |
| **WC** |  | |  | | | | | | | | | | | |
| Intercept | 3.6324 | 0.0002 | 1.9109 | <.0001 | 1.2815 | 0.1505 | 4.7332 | <.0001 | 1.8270 | 0.1333 | 2.4879 | 0.0430 | 1.0917 | 0.0038 |
| Time (5 y) | 0.009810 | 0.7305 | 0.09700 | <.0001 | -0.09499 | 0.0002 | 0.1449 | <.0001 | 0.04171 | 0.7969 | -0.02893 | 0.6276 | 0.002261 | 0.8983 |
| WC (≥90^th^ percentile) | 0.06425 | 0.5252 | -0.01897 | 0.6959 | 0.05068 | 0.5727 | 0.2271 | 0.0012 | -0.03945 | 0.4980 | 0.2569 | 0.1197 | 0.05747 | 0.2689 |
| WC*time | 0.05675 | 0.5896 | -0.00897 | 0.8665 | 0.03231 | 0.7260 | -0.06823 | 0.4310 | 0.2588 | 0.1862 | -0.02902 | 0.8846 | 0.09243 | 0.1376 |
| **BMI** |  | |  | | | | | | | | | | | |
| Intercept | 3.6158 | 0.0002 | 1.8880 | <.0001 | 1.2755 | 0.1534 | 4.7772 | <.0001 | 1.5010 | 0.2108 | 2.5127 | 0.0431 | 1.1119 | 0.0030 |
| Time (5 y) | -0.00484 | 0.8728 | 0.08948 | <.0001 | -0.1058 | <.0001 | 0.1500 | <.0001 | -0.05717 | 0.3410 | -0.01849 | 0.7619 | 0.009391 | 0.6166 |
| BMI (owt) | -0.01427 | 0.8473 | -0.01039 | 0.7723 | -0.01237 | 0.8506 | 0.1749 | 0.0009 | 0.06291 | 0.6125 | 0.2487 | 0.0508 | -0.01396 | 0.7203 |
| BMI (class I obesity) | 0.01599 | 0.8823 | 0.03158 | 0.5375 | 0.008962 | 0.9259 | 0.1697 | 0.0204 | 0.4754 | 0.0079 | 0.2918 | 0.1104 | 0.01866 | 0.7315 |
| BMI (class II obesity) | -0.02269 | 0.9223 | -0.2786 | 0.0154 | 0.06707 | 0.7442 | 0.3152 | 0.0680 | 1.3531 | 0.0005 | 0.02261 | 0.9544 | 0.4691 | 0.0003 |
| BMI (owt)*time | 0.1516 | 0.1218 | 0.06827 | 0.1629 | 0.1141 | 0.1856 | -0.03497 | 0.6444 | 0.2614 | 0.1324 | -0.1179 | 0.5057 | -0.03914 | 0.4801 |
| BMI (class I obesity)*time | 0.1372 | 0.2628 | 0.02212 | 0.7215 | 0.06651 | 0.5356 | -0.04488 | 0.6549 | 0.08921 | 0.6995 | 0.2702 | 0.2516 | 0.08350 | 0.2494 |
| BMI (class II obesity*time) ^±^ | – | – | – | – | – | – | – | – | – | – | – | – | – | – |
| **PBF** |  | |  | | | | | | | | | | | |
| Intercept | 3.5827 | 0.0002 | 1.8579 | <.0001 | 1.2603 | 0.1590 | 4.782 | <.0001 | 1.8698 | 0.1235 | 2.5876 | 0.0358 | 1.1349 | 0.0028 |
| Time | 0.006568 | 0.8195 | 0.09336 | <.0001 | -0.09501 | 0.0002 | 0.1535 | <.0001 | -0.03845 | 0.5088 | -0.04979 | 0.4074 | 0.005081 | 0.7774 |
| PBF (≥90^th^ percentile) | -0.02150 | 0.7820 | -0.01064 | 0.7821 | -0.01335 | 0.8455 | 0.1281 | 0.0280 | 0.08110 | 0.5668 | 0.01890 | 0.8964 | -0.01311 | 0.7578 |
| PBF*time | 0.1392 | 0.1753 | 0.02834 | 0.5857 | 0.06189 | 0.4913 | -0.01970 | 0.8106 | 0.4228 | **0.0281** | 0.3727 | 0.0598 | 0.1109 | 0.0630 |

WHtR = waist-to-height ratio (WHtR <.50 = reference group), WC = waist circumference (WC <90^th^ percentile = reference group), BMI = body mass index (BMI percentile – normal weight = reference group), PBF = percent body fat (PBF (<90^th^ percentile = reference group), Time-3 = reference group, E = regression estimate, p = p-value, y = years.

All analysis were adjusted for sex, time, ethnicity, annual household income, gestational age, maternal smoking during pregnancy, birth weight, and physical activity levels of the child.

^±^Missing values for class two obesity and time interaction as no child was identified to have class II obesity under 5 years of age.

Note: Infants provided non-fasting samples, while fasting samples were obtained from children aged 3 to 5 years, so birth measures were not included in the analysis.
